# Knowledge mapping of global trends for myasthenia gravis development: A bibliometrics analysis

**DOI:** 10.3389/fimmu.2023.1132201

**Published:** 2023-03-02

**Authors:** Fan Jiang, Yue Su, Ting Chang

**Affiliations:** ^1^ Department of Neurology, Tangdu Hospital, The Fourth Military Medical University, Xi’an, China; ^2^ The Second Brigade of Cadet, Basic Medical School, Air Force Military Medical University, Xi’an Shaanxi, China

**Keywords:** myasthenia gravis, bibliometric, Citespace, VOSviewer, Bibliometrix

## Abstract

**Background:**

Myasthenia gravis (MG) is an autoimmune disease with acquired neuromuscular junction transmission disorders. In the last two decades, various pathogenesis, application of immunosuppressive agents, and targeted immunotherapy have been significant events. However, extracting the most critical information from complex events is very difficult to guide clinical work. Therefore, we used bibliometrics to summarize and look forward.

**Methods:**

Science Citation Index Expanded (SCI-E) from the Web of Science Core Collection (WoSCC) database was identified as a source of material for obtaining MG-related articles. Scimago Graphica, CiteSpace, VOSviewer, and bibliometrix were utilized for bibliometric analysis. Knowledge network graphs were constructed and visualized; countries, institutions, authors, journals, references, and keywords were evaluated. In addition, GraphPad Prism and Microsoft Excel 365 were applied for statistical analysis.

**Results:**

As of October 25, 2022, 9,970 original MG-related articles were used for the bibliometric analysis; the cumulative number of citations to these articles was 236,987, with an H-index of 201. The United States ranked first in terms of the number of publications (2,877) and H-index (134). Oxford has the highest H-index (67), and Udice French Research University has the highest number of publications (319). The author with the highest average number of citations (66.19), publications (151), and H-index (53) was Vincent A. 28 articles have remained in an explosive period of citations. The final screening yielded predictive keywords related to clinical trials and COVID-19.

**Conclusion:**

We conducted a bibliometric analysis of 9,970 original MG-related articles published between 1966 and 2022. Ultimately, we found that future MG research hotspots include two major parts: (1) studies directly related to MG disease itself: clinical trials of various targeted biological agents; the relationship between biomarkers and therapeutic decisions, pathogenesis and outcome events, ultimately serving individualized management or precision therapy; (2) studies related to MG and COVID-19: different variants of COVID-19 (e.g., Omicron) on MG adverse outcome events; assessment of the safety of different COVID-19 vaccines for different subtypes of MG.

## 1 Introduction

Myasthenia gravis (MG) is a low-incidence disease, with an estimated incidence of 0.3–2.8 per 100,000 person-years worldwide (1). The mortality rate of patients with MG has decreased yearly with the advent of therapeutic drugs (2). However, there remains a management gap in this disease, as patients may have to wait several years for an accurate diagnosis and often experience an unpredictable clinical course, including stabilization, remission, relapse, and exacerbation (3–11).

MG is often overlooked, especially in elderly patients, due to the ease of misdiagnosis as other diseases (3). An epidemiological study (N=100) found that 26% and 13% of patients with MG had a 2-year and a 5-year delay in diagnosis, respectively (4). Consequently, delayed diagnosis may increase unnecessary examinations and treatments, increasing the disease burden of patients; similarly, it may cause delayed treatment, i.e., patients not receiving rapid and timely treatment, leading to disease progression. For example, ocular MG (OMG) may transform into generalized MG (GMG) or even myasthenic crisis, causing poor prognostic outcomes. Besides the diagnosis delay difficulties, there are also many challenges in the management of the disease: 1) many treatment regimens have variable research conclusions (7–9); 2) many treatment regimens have reported treatment-related adverse effects (7, 10, 12); 3) the use of some treatment regimens may be limited due to comorbidities (12); and 4) serological profile of MG may affect the response to treatment (9, 10).

The above reservations were raised based on a summary of our previous work. Although the main contradictions might have been widely understood, all MG-related knowledge grooming, for the moment, is primarily derived from the accumulated experience of the discipline leaders. The quality of reviews and systematic reviews published by academic leaders is undoubtedly guaranteed. However, the accumulated knowledge and analysis of the leaders require extended time for precipitation for such in-depth and comprehensive reviews. Just as the Nobel Prize winners were awarded much later than their research results were made public (13), time is the only criterion to test the truth. Therefore, high-quality, comprehensive reviews are always lacking, and this disadvantage is precisely remedied by bibliometrics because its ease of operation significantly reduces the threshold for complete reviews.

Meanwhile, burst detection with predictive function is an algorithm developed by Kleinberg, capable of identifying research frontiers (14). The vast discipline of medicine needs to make full use of tools that combine retrospective summarization and prospective prediction functions to deeply explore the literature’s characteristics from the big data level. Bibliometrics is an example of such a tool. When knowledge mapping is combined with the truth of scientific practice, the presented results can compensate for the summary reviews performed by empirical accumulation. After summarizing, predicting future research directions through scientific algorithms is a more scientific approach than the researcher’s intuition, and this is where the significance of bibliometric analysis lies. This paper aimed to review 9,970 MG-related articles in the past 56 years and analyze the seven aspects of the overall status of the discipline, country, institution, author, journal, literature, and keywords. A burst analysis will be conducted on the literature and keywords to indicate the literature that should be focused on in current research and identify the possible directions for future research.

To our knowledge, the first application of bibliometrics in the field of MG was finished by our team members (15), and this article is the first study to provide a comprehensive bibliometric analysis for MG.

## 2 Materials and methods

### 2.1 Data materials

Science Citation Index Expanded (SCI-E) of the Web of Science Core Collection (WoSCC) database was selected for analysis for the following reasons: 1) SCI-E provided the document pattern needed for bibliometric analysis software such as CiteSpace, VOSviewer, Scimago Graphica, and Bibliometrix, and 2) SCI-E database is the most authoritative and highest standard global database with broad usage. The MG research originated from T Buzzard’s work conducted 120 years ago (16). However, early publications on MG were minimal due to poor publishing and information technology, and the data recorded in the database did not significantly increase until the middle of the twentieth century. In addition, the upper limit of the number of articles analyzed by VOSviewer and CiteSpace softwares and SCI-E database citation reports is 10,000. Therefore, articles published between 1966 and 2022 were included in this study.

### 2.2 Methods

#### 2.2.1 Retrieval strategies

**Figure 1:**
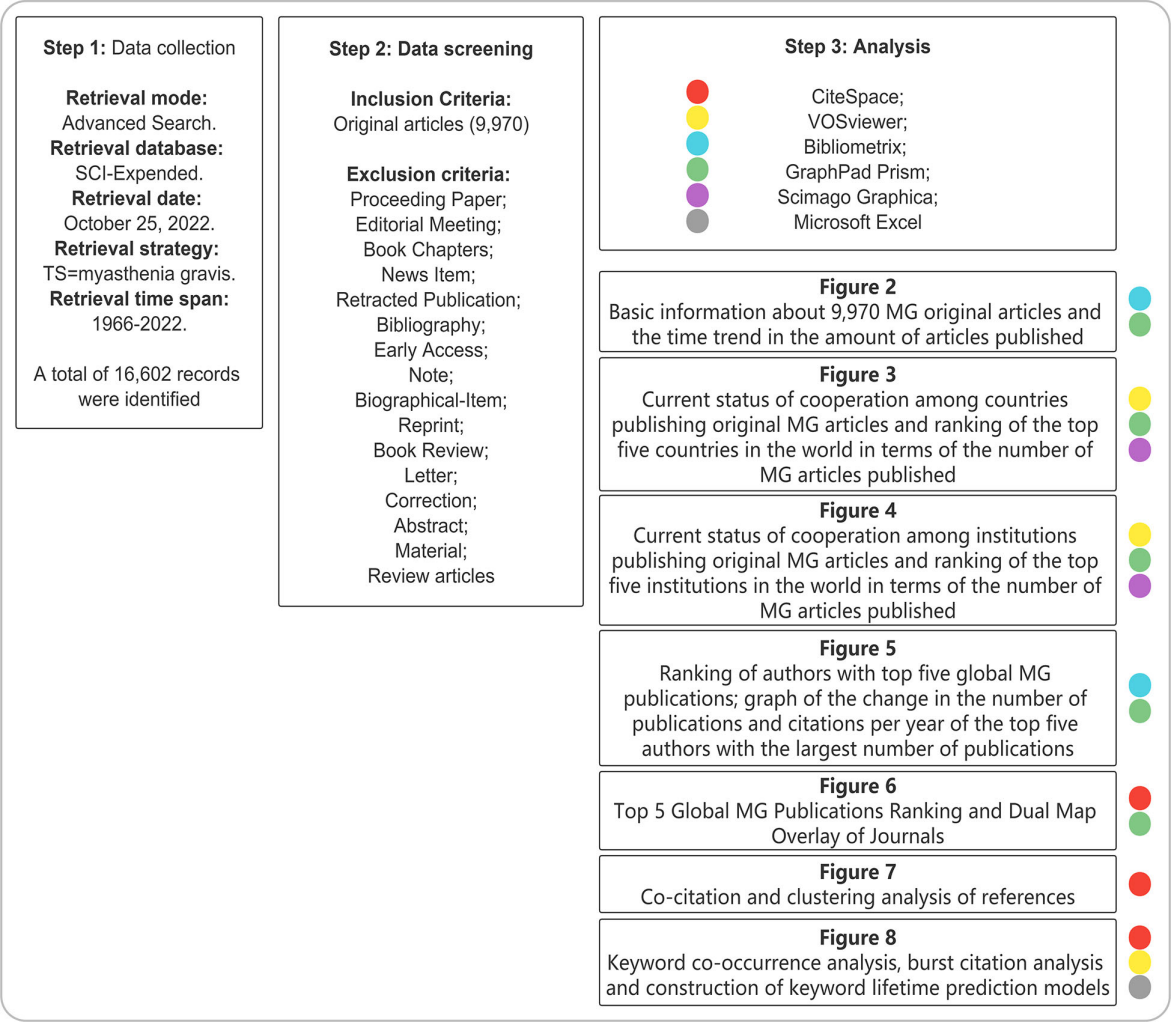
Retrieval strategy.

This paper used advanced retrieval functions to improve the quality of the retrieved information, and we selected Thematic Suffix (TS) for retrieval. Specific search rules are as follows: (1) TS =(MG); (2) A total of 16,602 MG-related records from 1966 to 2022 were retrieved (searched on October 25, 2022). Eligible resources were limited to original articles; (3) All content, including title, author, abstract, keywords, and cited literature, were recorded; (4) The selected documents were output in the form of “full-text records and citations” for further analysis. Since the CiteSpace and VOSviewer could approve only the txt version, these text files were renamed “Download*.txt.” Finally, the literature screening process is shown in **Figure 1**.

#### 2.2.2 Data extraction

The researchers imported the data into Microsoft Excel 365 (Microsoft, Raymond, Washington, USA) for further processing. Two researchers (FJ and YS) independently conducted data extraction and literature selection and analysis to ensure the reliability of the results. The two researchers discussed and reached an agreement in cases of disagreements, and unresolved differences were resolved through a third-party (TC) consultation. The report automatically generated by SCI-E showed the number of publications, average citation, and H-index. This paper mainly analyzed countries, institutions, authors, and journals based on the indexes above.

Meanwhile, references and keywords were mainly analyzed for centrality and burst strength. The H-index is calculated from h papers published by a scientist/country, each of which is cited at least h times (17, 18). This index is often used to assess the scientific research influence and productivity of researchers/countries.

#### 2.2.3 Data visualization and analysis

Softwares (CiteSpace [6.1.R6], VOSviewer [1.6.18], and Bibliometrix) were used to manufacture the knowledge network map, and GraphPad Prism and Microsoft Excel 365 for statistical analysis.

#### 2.2.4 CiteSpace

CiteSpace (Version 6.1.R6, downloaded from https://sourceforge.net/projects/citespace/) is a computer program developed by Professor Chen based on Java language, famous for highly influential visualization software. This software can obtain quantitative information and discover relevant developments and trends in specific scientific research fields with loaded burst and cluster analyses mode (19–21).

#### 2.2.5 VOSviewer

VOSviewer (version 1.6.18, Holland, downloaded from http://vosviewer.com) is a software tool originally jointly developed by Ike and Waltman from Leiden University based on the JAVA platform for constructing and visualizing bibliometric networks (22). These networks may include journals, researchers, or individual publications, and they can be built based on citation, bibliographic coupling, co-citation, or co-authorship relations (23).

#### 2.2.6 Bibliometrix

Bibliometrix (download package Bibliometrix 4.0.1; https://www.bibliometrix.org/home/) is a package created and developed by Massimo Aria and Corrado Cuccurullo for the R statistical programming language (R Studio software 2022.07.2 (R version 4.2.1 (2022-06-23 UCRT)) for quantitative research in scientometrics and bibliometrics (24).

#### 2.2.7 Statistical analysis

Statistical analysis and scientific mapping were performed using GraphPad Prism 9 (San Diego, CA, USA; https://www.graphpad.com/scientific-software/prism), Scimago Graphica (https://graphica.app/), and Microsoft Excel 365 (Microsoft Corporation; https://www.microsoft.com/en-us/microsoft-365/excel). Metrological analysis software/servers such as CiteSpace, Bibliometrix, and VOSviewer support the processing of information in English only. Message text containing other language types is automatically excluded during analysis.

## 3 Results

### 3.1 Search results and the status of MG research

**Figure 2:**
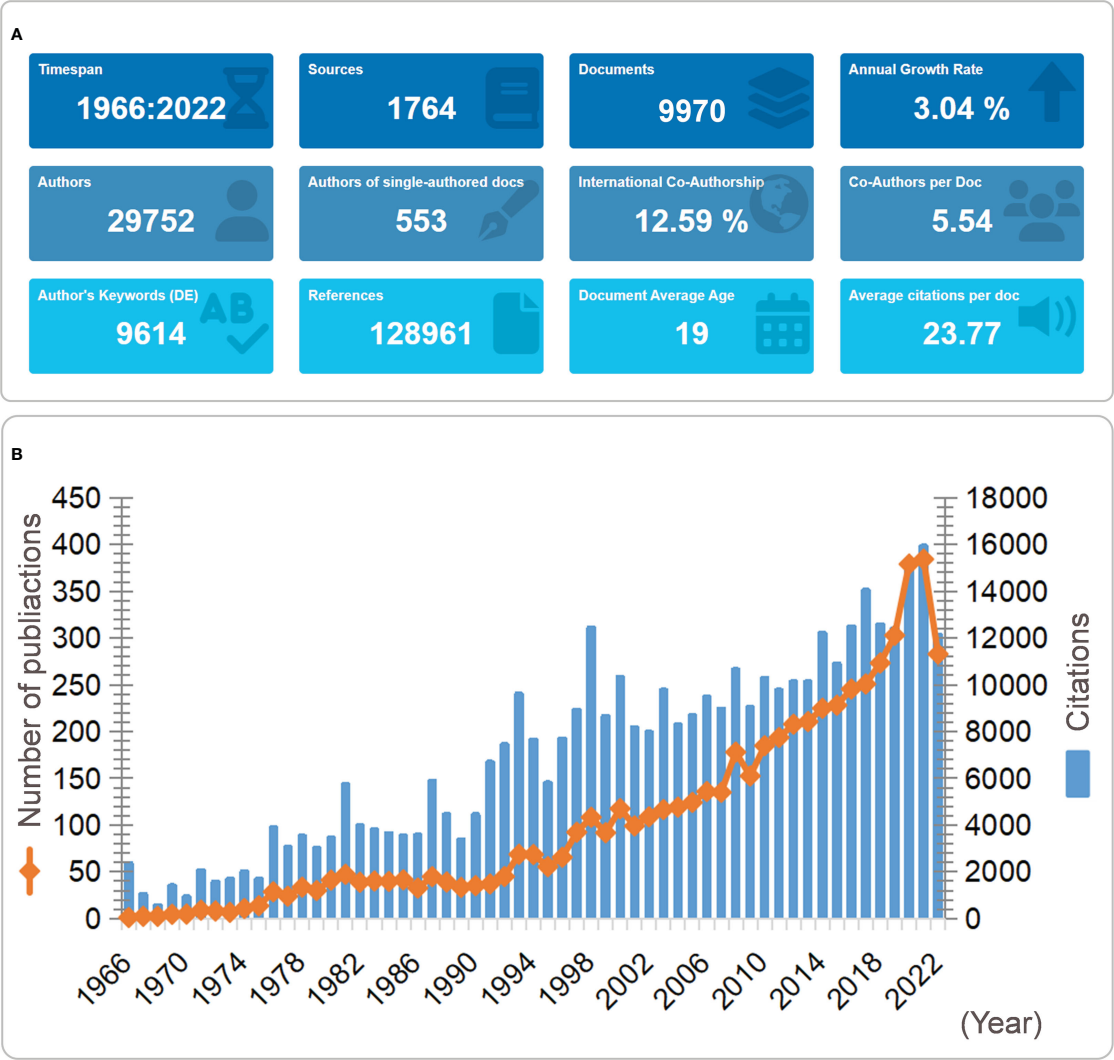
**(A)** Basic information of 9970 MG-related articles included; **(B)** Overall publication trends and citations. **(A)** The period of included articles, the number of journal categories, the total number of articles, the annual growth rate, the total number of authors, the number of articles published by a single author, the proportion of international co-authors, the number of co-authors of an article, the keywords given by the author, the number of references cited, the average life span of each article, the average number of citations per article. **(B)** The figure shows the overall number of posts and citations in the MG field from 1966 to 2022.

The search strategy in **Figure 1** was implemented, and 16,602 results were retrieved, including 9,970 original research articles. The current status of MG research was described using Bibliometrix in **Figure 2A**. Our analysis reviewed 1966–2022, and 1,764 journals were included. The search result included 9,970 articles with an annual publication growth rate of 3.04%. There were 29,752 authors, and a single author wrote 553 articles. Authors with international cooperation accounted for 12.59%. Each article had an average of 5–6 authors; 9,614 keywords were provided, and 128,961 references were cited. The average life span of each paper from being noticed to being unknown was 19 years; each article had been awarded an average of 23–24 times.

### 3.2 The overall trend in the number of publications and citations

From 1966 to 2022, the number of papers published steadily increased from single digits to more than 400 in 2021 (**Figure 2B**). The number of citations was consistent with the trend of the number of published papers, and noteworthily, there were apparent peaks in the number of citations in 1994 and 1999, which might be closely related to the vital breakthroughs in the field of MG during this period (**Figure 2B**).

### 3.3 Current status of national research output and international cooperation

**Figure 3:**
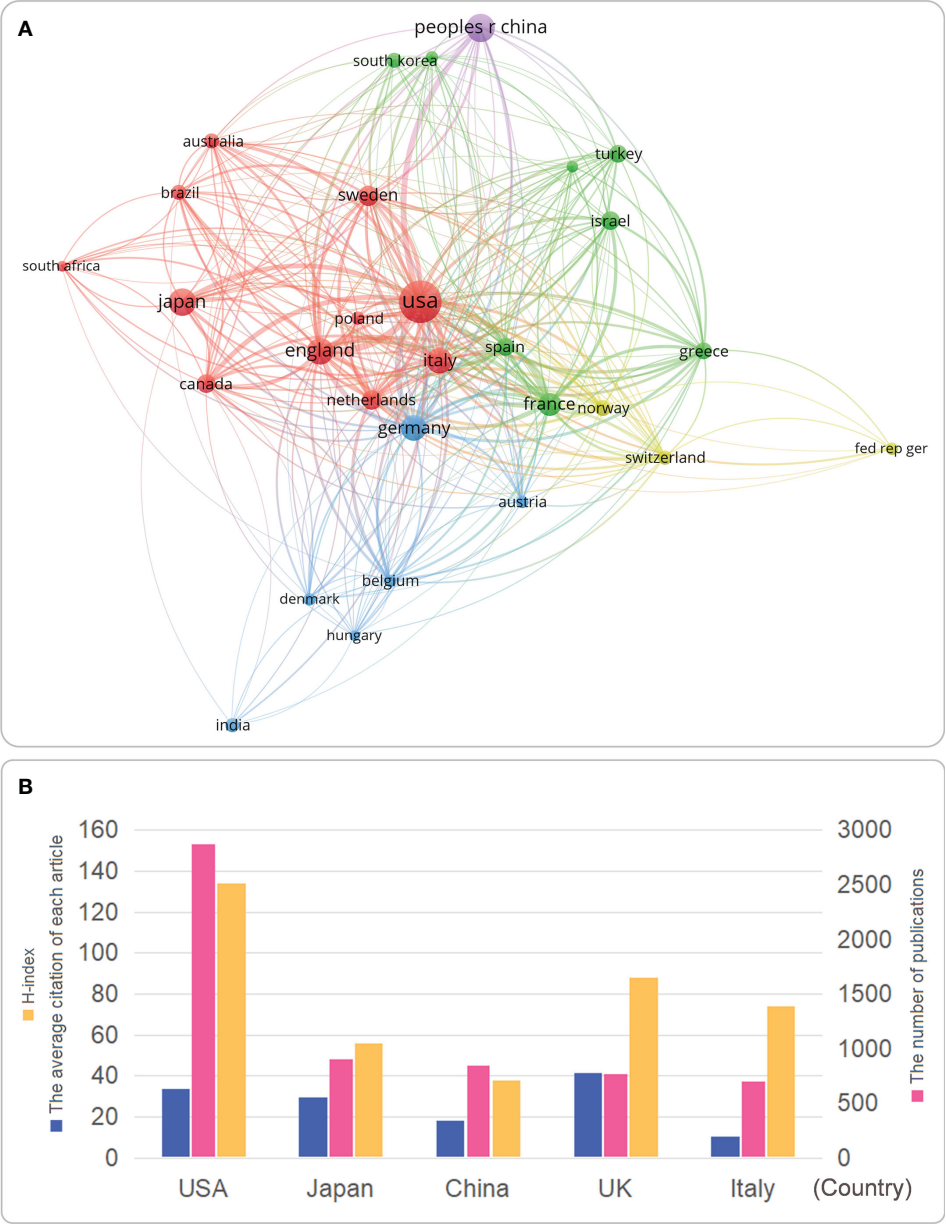
**(A)** Visual maps of international cooperation between countries studying MG; **(B)** countries in the top five international rankings in MG studies. **(A)** the proportional relationship between the size of the ball and the number of publications is shown in the corner label. Connection indicates that there is a cooperative relationship. Countries that conduct MG studies around the world are divided into four partner groups according to the co-authors of 9970 articles; **(B)** countries ranked in the top five in the world are evaluated according to the number of publications, the H index, and the average number of citations per article.

The txt file of 9,970 MG-related articles was imported into VOSviewer to generate a country cooperation network map. Parameters were set for 30 countries with at least 49 publications out of 106 countries to be included in further analysis. First, the selected 30 countries were divided into five clusters according to the degree of cooperation: Cluster 1 (red) mainly included Australia, Brazil, Canada, UK, Italy, Japan, Netherlands, Poland, South Africa, Sweden, and the USA; Cluster 2 (green) mainly included Czech Republic, France, Greece, Israel, UK, South Korea, Spain, and Turkey; Cluster 3 (blue) included Austria, Belgium, Denmark, Germany, Hungary, and India; Countries in cluster 4 were Germany, Norway, and Switzerland; Cluster 5 (purple) included China (**Figure 3A**). Second, USA, UK, China, Italy, and Japan had a large number of publications and were represented by a larger area of the circle on the figure (**Figure 3A**). Therefore, we sorted out the top five countries in terms of publications and presented them in **Figure 3B**. 1) The number of publications: USA (2,877) > Japan (905) > China (845) > UK (767) > Italy (697); 2) The average citation of each article: UK (41.4) > USA (33.86) > Italy (29.44) > Japan (18.06) > China (10.59); and 3) H-index: USA (134) > UK (88) > Italy (74) > Japan (56) > China (38).

### 3.4 Output and collaboration status of MG research institutions

**Figure 4:**
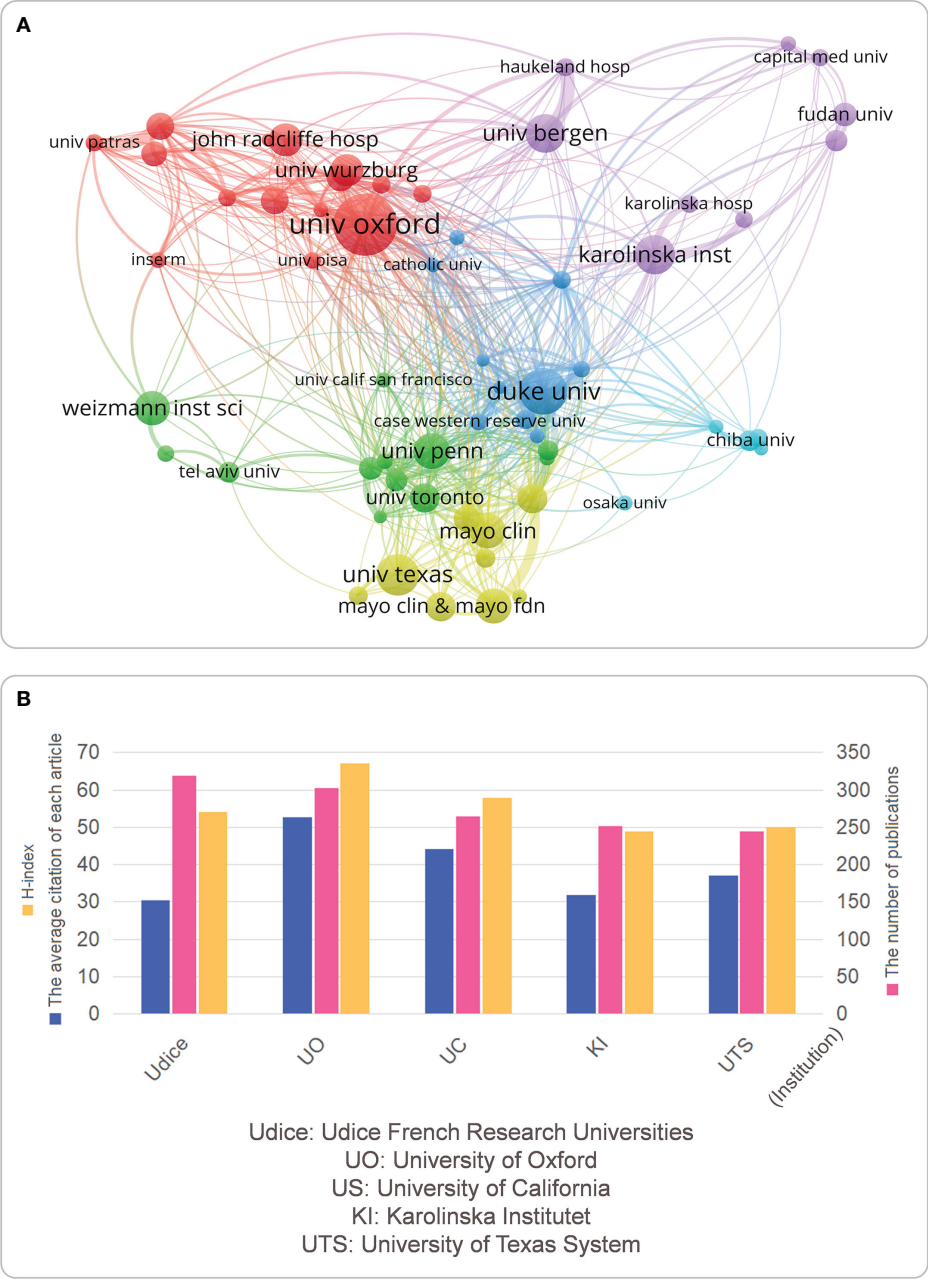
**(A)** a visual map of the current status of inter-agency cooperation in MG research; **(B)** the top five institutions worldwide in the field of MG research. **(A)** Different colors represent different cooperative groups, the connection indicates cooperation, and the connection width indicates the close degree of cooperation; **(B)** Institutions ranked international top 5 related to MG are evaluated according to the number of publications, H index and the average number of citations per article.

A total of 9,970 articles were selected and imported into VOSviewer, and the parameter was set as an institution to publish at least 39 articles. Among the 6,120 institutions, 60 met the inclusion criteria. The 60 institutions were divided into six clusters according to the degree of collaboration: 1) The collaboration groups represented by Oxford University, Wolzburg University, and John Radcliffe Hospital were mainly represented by the red cluster; 2) The cooperation groups represented by the green cluster were mainly Weizmann Institute of Science, the University of Pennsylvania and the University of Toronto; 3) The collaboration groups represented by the yellow cluster were mainly Mayo Clinic & Mayo Foundation for Medical Education and Research and the University of Texas; 4) The cooperation groups represented by the blue cluster were Duke University and Case Western Reserve University; 5) The cooperation groups represented by the purple cluster were mainly University of Bergen, Karolinska Institutet, Haukeland University Hospital, Fudan University, and Capital Medical University; 6) The cooperative organizations represented by the cyan cluster were mainly Chiba University and Osaka University (**Figure 4A**). To further discover the specific publications of the institutions studying MG, we presented the top five institutions according to the number of publications in **Figure 4B**. 1) In terms of the number of publications: Udice French Research Universities (319) > University of Oxford (303) > University of California (264) > Karolinska Institutet (251) > the University of Texas System (244); 2) the average number of citations per article: University of Oxford (52.7) > University of California (44.08) > the University of Texas System (36.97) > Karolinska Institutet (31.88) > Udice French Research Universities (30.44); and 3) H-index: University of Oxford (67) > University of California (58) > Udice French Research Universities (54) > the University of Texas System (50) > Karolinska Institutet (49).

### 3.5 Current status of publications and research careers of the top five global authors

**Figure 5:**
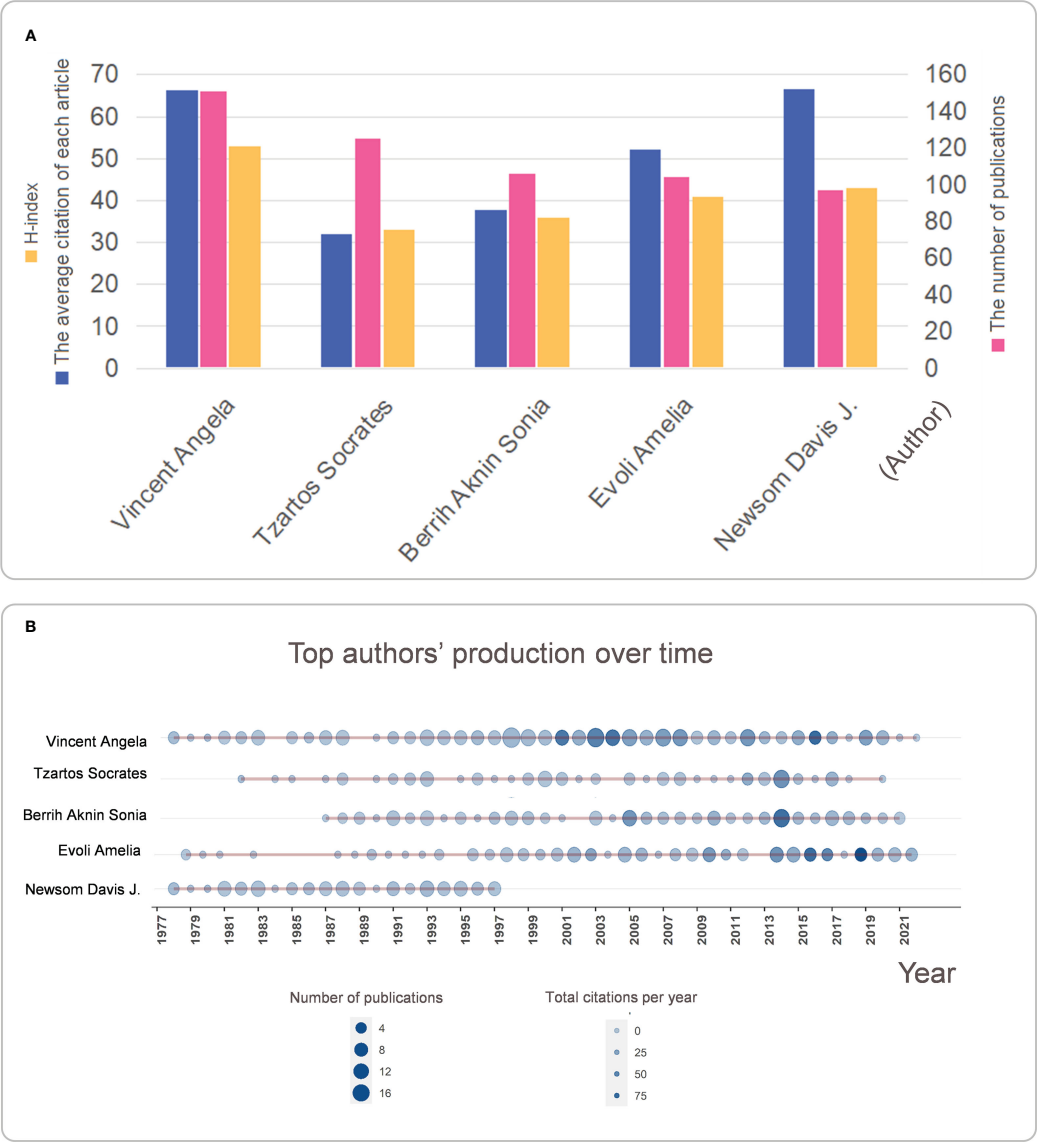
**(A)** The top five MG researchers ranked in terms of the number of publications; **(B)** A visual view of the world’s publishing career of the top five MG researchers. **(A)** The top five international MG researchers are evaluated in terms of the number of publications, the H-index, and the average number of citations per article; **(B)** The number of articles published each year and the average amount of citations per year from 1977 to 2022 are shown in the time graph, through which the careers of the researchers can be seen directly.

Based on the basic information from the 9,970 articles, we enumerated the top five international scientists in the field of MG (**Figure 5A**). Number of publications: Vincent Angela (151) > Tzartos Socrates (125) > Berrih Aknin Sonia (106) > Evoli Amelia (104) > Newsom Davis J. (97); Average number of citations per article: Newsom Davis J. (66.42) > Vincent Angela (66.19) > Evoli Amelia (52.06) > Berrih Aknin Sonia (37.72) > Tzartos Socrates (32); and H-index: Vincent Angela (53) > Newsom Davis J. (43) > Evoli Amelia (41) > Berrih Aknin Sonia (36) > Tzartos Socrates (33). To further analyze the top five MG researchers, we assessed these five authors’ annual publications and annual citations from 1977 to 2022 (**Figure 5B**). The scientific research output of Vincent Angela has been relatively stable since 1977. She published a dozen papers per year from 1990 to 2020, and the number of citations for articles produced during this period exceeded 50. The scientific research output of Tzartos Socrates has been distributed discontinuously since 1982. Since 2005, the scholar’s paper output has gradually stabilized at eight papers per year, with more than 50 citations. Berrih Aknin Sonia has published a very stable number of articles every year since 1987 and has done exceptionally well in the 20 years since 2003, with a relatively high number of articles published and the number of citations per year. The output of Evoli Amelia from 1977 to 1995 was pretty unstable, and the number of citations in this period was relatively low. However, since 1995, especially in 2018, the work has attracted extensive attention. Newsom Davis J. was quite active in publishing papers from 1977 to 1997, and the number of citations per year in this period was also stable at about 30. However, the number of articles published after the 21st century might be too small to be shown in the figure. This phenomenon might be explained by the retirement of Professor Newsom Davis J. from Oxford University in 1998.

### 3.6 Current status of publication and distribution of journals

**Figure 6:**
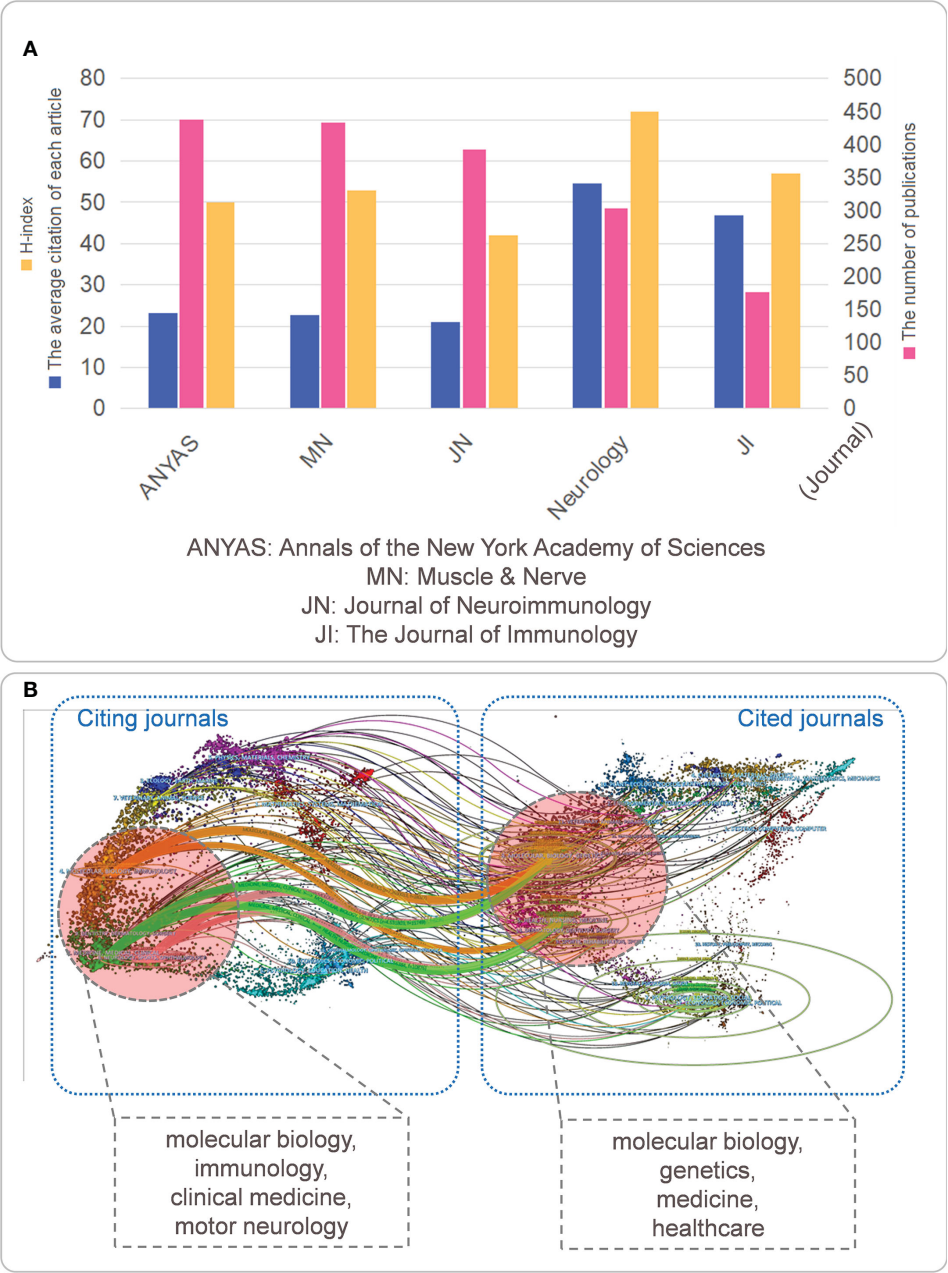
**(A)** The five journals with the most significant number of publications; **(B)** Double-picture superposition atlas of periodicals. **(A)** The five journals with the most significant number of articles related to MG are evaluated in terms of the H-index and the average number of citations per article as well; **(B)** On the left is the leading distributor of MG-related journals (citing journals); on the right is the disciplines where the journals cited by MG articles are located (cited journals). The connection represents the subject knowledge transfer path.

When countries, institutions, and individuals had extraordinary work done, the results were presented in journals, and it was necessary to analyze the publication and distribution of the journals. The top five journals with the most significant number of publications are shown in **Figure 6A**. The number of publications: *Annals of the New York Academy of Sciences* (438) > *Muscle & Nerve* (434) > *Journal of Neuroimmunology* (392) > *Neurology* (304) > *The Journal of Immunology* (177); Average number of citations per article: *Neurology* (54.63) > *The Journal of Immunology* (46.86) > *Annals of the New York Academy of Sciences* (23.27) > *Muscle & Nerve* (22.71) > *Journal of Neuroimmunology* (20.99); and H-index: *Neurology* (72) > *The Journal of Immunology* (57) > *Muscle & Nerve* (53) > *Annals of the New York Academy of Sciences* (50) > *Journal of Neurology Neuroimmunology* (42). In addition, we conducted double map coverage of journals for MG to explore further the topic distribution of journals and the transfer path of disciplinary knowledge (**Figure 6B**). Three enlarged areas: 1) The map of cited journals on the left: represented journals with published research focused on four main fields, molecular biology, immunology, clinical medicine, and motor neurology; 2) On the right was a map of cited journals that focused on molecular biology, genetics, medicine, and healthcare.

### 3.7 Content distribution and time variation of references

**Figure 7:**
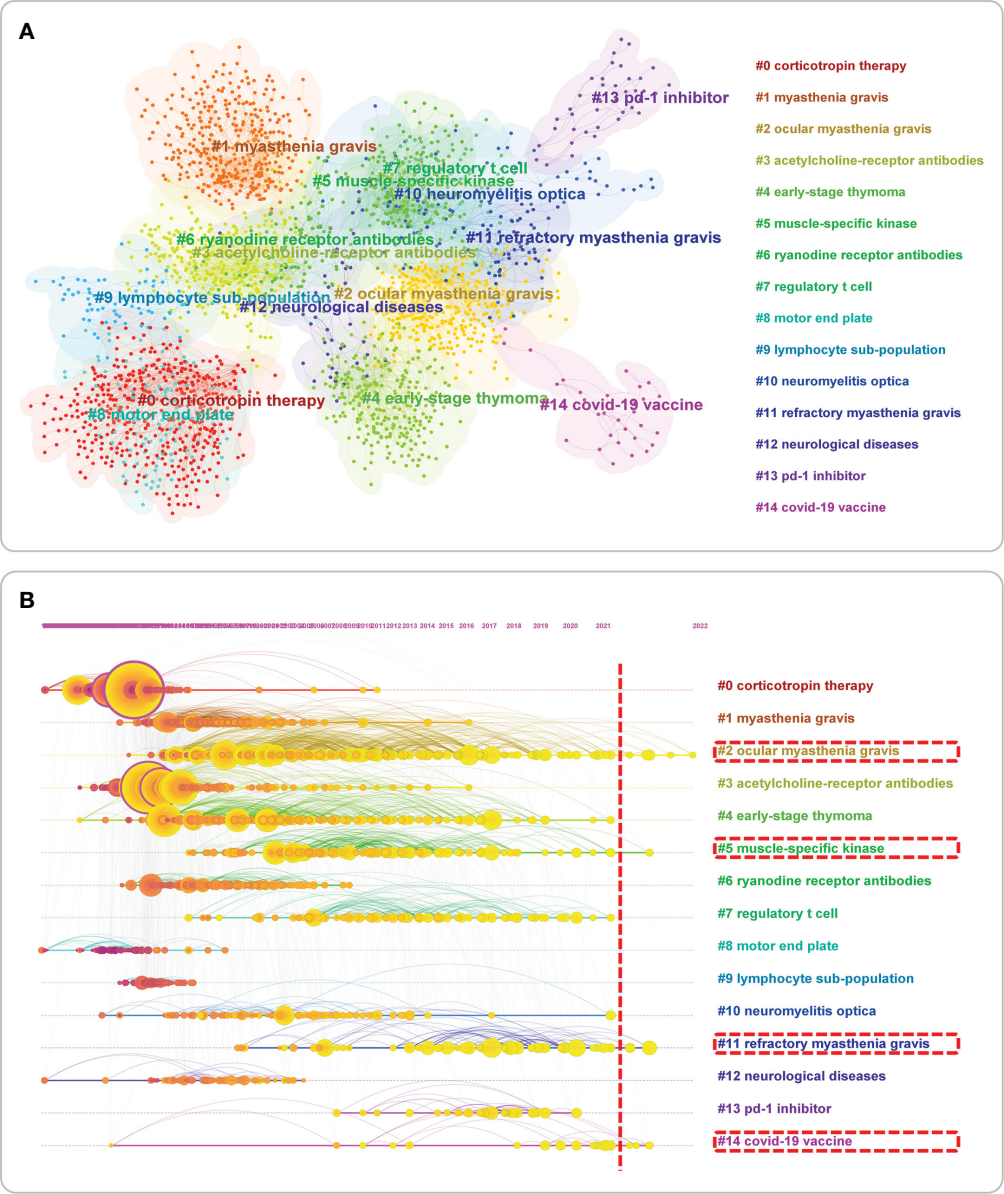
**(A)** Co-citation and cluster analysis of references; **(B)** Clustering analysis on the timeline view. **(A)** The co-citation of references reflects which articles were studied in the same direction. Cluster analysis classifies the references related to MG in the past 60 years into four clusters using the maximum likelihood ratio(LLR) method. Combined with the calculation of the degree of centrality and the transfer of references with high centrality on the time axis, we can analyze how the research process of the discipline changes and what representative references are available at each stage; **(B)** Circles indicated references; the size of the diameter of the circle was closely related to the number of citations; those with purple circles stated the existence of significant turning points in scientific knowledge, i.e., higher centrality; the purple axis indicated 1966-2022; yellow circles still to the right of the red vertical line indicated the existence of relevant literature; the content displayed by the red wireframe was hot.

When journals can grasp the diffusion of knowledge from the perspective of multiple disciplines, the literature captures the distribution of different content sub-categories from a single discipline’s perspective. The co-citation analysis can help us classify the literature and understand the current research field’s main components according to the clusters’ characteristics. Therefore, the co-citation of the literature is presented in **Figure 7A**, and the literature could be roughly divided into 20 parts. After understanding the research content’s composition, the research content’s evolution process naturally needed to be answered. Therefore, we conducted clustering and centrality analysis of the literature through CiteSpace to determine the main research content of each period from 1966 to 2022 (**Figure 7B**), and the detailed clustering and co-citation analysis results are presented in **S1**. Finally, considering that the 10 most cited articles were also of interest to most MG researchers, they are listed in **Table 1**.

**Table 1:** Top 10 papers with highest citations.

No.	Title	Author	Published time	Journal	2018	2019	2020	2021	2022	Average citations per year	Total
1	ANTIBODY TO ACETYLCHOLINE-RECEPTOR IN MYASTHENIA-GRAVIS - PREVALENCE, CLINICAL CORRELATES, AND DIAGNOSTIC VALUE	LINDSTROM, JM; SEYBOLD, ME; (…); DUANE, DD	1976	Neurology	16	17	31	31	16	26.55	1248
2	A RANDOMIZED TRIAL COMPARING INTRAVENOUS IMMUNE GLOBULIN AND PLASMA-EXCHANGE IN GUILLAIN-BARRE-SYNDROME	VANDERMECHE, FGA and SCHMITZ, PIM	1992	New England Journal of Medicine	16	19	15	10	18	30.55	947
3	A role for humoral mechanisms in the pathogenesis of Devic’s neuromyelitis optica	Lucchinetti, CF; Mandler, RN; (…); Lassmann, H	2002	Brain	33	43	36	48	33	41.52	872
4	Nivolumab alone and nivolumab plus ipilimumab in recurrent small-cell lung cancer (CheckMate 032): a multicentre, open-label, phase 1/2 trial	Antonia, SJ; Lopez-Martin, JA; (…); Calvo, E	2016	Lancet Oncology	137	152	157	161	63	115.86	811
5	Auto-antibodies to the receptor tyrosine kinase MuSK in patients with myasthenia gravis without acetylcholine receptor antibodies	Hoch, W; McConville, J; (…); Vincent, A	2001	Nature Medicine	41	36	51	37	29	35.77	787
6	Potassium channel antibody-associated encephalopathy: a potentially immunotherapy-responsive form of limbic encephalitis	Vincent, A; Buckley, C; (…); Palace, J	2004	Brain	29	36	22	17	16	40.47	769
7	Anti-inflammatory activity of human IgG4 antibodies by dynamic Fab arm exchange	Kolfschoten, MV; Schuurman, J; (…); Parren, PWHI	2007	Science	52	46	54	39	29	40.38	646
8	NEUROMUSCULAR JUNCTION IN MYASTHENIA-GRAVIS - DECREASED ACETYLCHOLINE RECEPTORS	FAMBROUGH, DM; DRACHMAN, DB and SATYAMURTI, S	1973	Science	4	8	6	7	1	11.48	574
9	THE INFLUENCE OF ANTIGEN ORGANIZATION ON B-CELL RESPONSIVENESS	BACHMANN, MF; ROHRER, UH; (…); ZINKERNAGEL, RM	1993	Science	27	29	30	32	15	18.53	556
10	The epidemiology of autoimmune diseases	Cooper, GS and Stroehla, BC	2003	Autoimmunity Reviews	40	42	66	53	47	27.6	552
Accumulation	10 Publications				39.5	42.8	46.8	43.5	26.7	38.871	776.2
Total	9,970 Publications				10927	12111	15157	15367	11312	4138.86	235915
Ratio	0.10%				0.36%	0.35%	0.31%	0.28%	0.24%	0.94%	0.33%

### 3.8 Possible future research trends were analyzed in three ways

The unique feature of bibliometrics is the capacity to predict future research hotspots. Although there are many methods to predict keywords, the effectiveness of various ways needs to be verified by statistical methods, which is also the biggest bottleneck of the published articles in bibliometrics. Therefore, we used three analysis methods to minimize the error of keyword analysis caused by different algorithms, parameters, and time slices.

The first method was to perform a co-occurrence analysis of keywords through VOSviewer. The parameter set was at least 71 occurrences of a keyword, and 100 of the 9,331 keywords reached the selection criteria. The results of the keyword clustering analysis were labeled as clusters 1 and 2 according to the temporal distribution, with cluster 1 representing the fundamental medical part of MG research that dominated before 2010, such as “T-cell induced γ-interferon release,” “alpha subunit,” “monoclonal antibody development,” and “*in vivo* mouse injection.” However, over time, current MG research focused on cluster 2: (1) standard, double-blind clinical trials of various new targeted drugs; (2) evaluation of outcome, prognosis, survival, risk factors, quality of survival, and other indicators based on typing.

**Figure 8:**
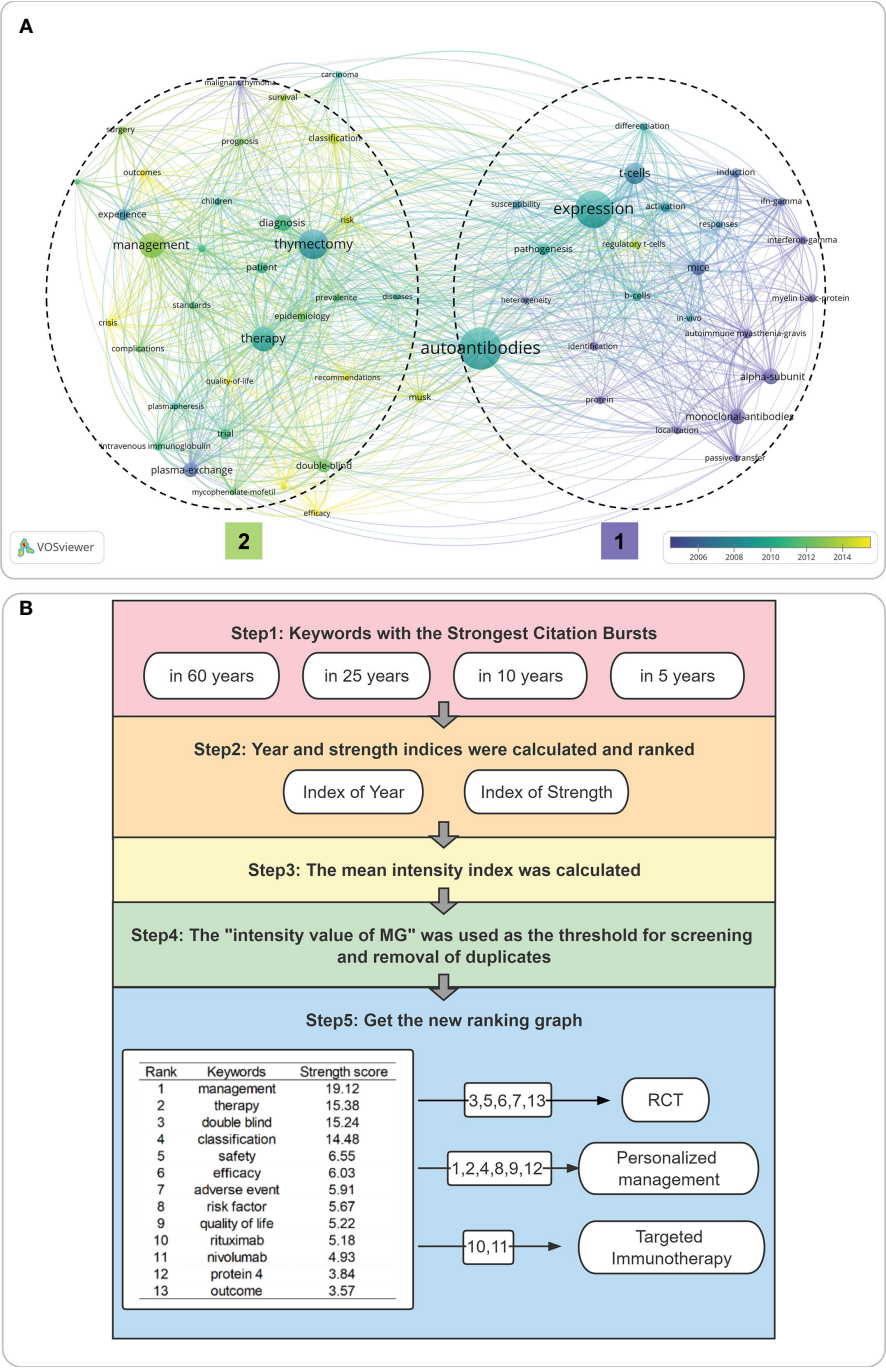
**(A)** Co-occurrence Analysis of keywords; **(B)** Keyword burst analysis presented after optimization **(A)** Visualize the activity situation of keywords based on VOSviewer. It is divided into two clusters according to the different beginning points of activity; **(B)** Through the processing steps shown in the figure, the keyword burst analysis results of other time slices are processed, and finally, the 13 keywords with the highest strength score are integrated. RCT referred to Randomized Controlled Trials.

The second approach benefitted from CiteSpace’s ability to select arbitrary time ranges for time slicing. We performed a burst citation analysis of keywords from four ranges (**Figure 8B**: 60 years, 25 years, 10 years, and 5 years). This measure avoided the bias of a single period for early predictive success. We followed the following steps for the predictive keyword results obtained for the different time slices: (1) Keywords with the most powerful citation bursts in 60/25/10/5 years were included;(2) Year and strength indices were calculated and ranked; (3) The mean intensity index was calculated; (4) The “intensity value of MG” was used as the threshold for screening and removal of duplicates; (5) Get the new ranking graph. Finally, 13 keywords were left, including (1) management; (2) therapy; (3) double-blind; (4) classification; (5) safety; (6) efficacy; (7) adverse event; (8) risk factor; (9) quality of life; (10) rituximab (RTX); (11) nivolumab; (12) protein 4; (13) outcome. Combining our team’s previously published “Knowledge mapping of targeted immunotherapy in myasthenia gravis from 1998 to 2022: a bibliometric analysis” and our team’s clinical practice results, we hypothesized that these 13 keywords encompass the following key research directions: (1) standard double-blind clinical trials to assess safety, efficacy and side effects; (2) biochemical marker-based precision typing to improve management and treatment options; and (3) marker-based precision typing for improved management and treatment options, and new targeted agents.

## 4 Discussion

To the best of our knowledge, this study is the first to use bibliometrics to measure the development trend of MG’s broad research field from 1966 to 2022. Unlike systematic and scope-defining evaluation, bibliometrics analysis has become a powerful tool to summarize the current knowledge situation and predict the future development trend based on the view of discipline. The visual map shows the results through digital technology based on information science, computer science, scientometrics, statistics, and applied mathematics. These results are specific knowledge structure domains and cluster structure relationships generated by VOSviewers or CiteSpace. After excluding 6,632 studies that did not meet the inclusion criteria, 1,764 journals, 9,970 papers, and 128,961 cited references from 6120 institutions in 106 countries were eligible for analysis. We used bibliometrics analysis techniques and visual analysis tools to analyze national regions, research institutions, journals, authors, references, and keywords to summarize the knowledge about MG and predict research hotspots in emerging topics.

From 1966 to 2022, the total number of MG-related articles was 16,602 (in WoSSC SCI-E). Although the starting time of growth is different, the number of published papers shows a continuous growth trend with time, whether in the field of MG or other neuroimmune diseases (**Figure 2**). The reasons for the increasing number of publications on MG and other neuroimmune diseases include: (1) although MG is a rare disease, the total prevalence of MG is 150-250 cases per million people and the annual incidence is assumed to be 8-10 cases per million people-years. In countries with large population bases, such as China and India, the tension between the urgent medical needs of patients with rare diseases and the large clinical burden and research burden is particularly acute (25); (2) the prevalence of MG is increasing annually, and therefore research in this field is gradually increasing; and (3) the field of neuroimmunology has developed rapidly. In recent years, remarkable achievements have been made in MG as a neuroimmune disease in various aspects, including extensive epidemiological surveys, exploration of etiology and pathogenesis, construction of *in vivo*/*in vitro* models, improvement of diagnosis and treatment technology, and development of targeted biological agents, all of which have provided a foundation for its development. As a result, the number of publications has been increasing yearly.

The molecular biological mechanism of disease-targeted immune therapies and biomarkers are essential to the current study hotspots in neuroimmune disorders. Most clinical treatment methods and basic research ideas are not applied to MG unless other neuroimmune disease research is mature, or a specific treatment has been used in the clinic. Therefore, the published output trend of MG can somewhat represent the development trend of neuroimmune disease research. In addition, the main reason for the presence of cluster label 10 (neuromyelitis optica [NMO]) in **Figure 7** is that both diseases, MG and NMO, belong to the same neuroimmune class of diseases, and they overlap in pathogenesis and therapeutic approaches to some extent.

The yearly increase in MG publications is due to the outstanding contributions of countries, institutions, scholars, and journals. Regarding countries/regions/institutions, the US is the leading country in publications and citations in this field (**Figure 3**), partly due to the nation’s long-standing foundation in the biomedical field and vital funding and sufficient research institutions and researchers in this field (26). Two of the top five institutions (the University of California and the University of Texas System) are from the US (**Figure 4**), so we concluded that this might be the reason for the rapid development of this field in the US. Second, we observed that China, as a developing country, has jumped to third place in terms of the number of publications (**Figure 3**), indicating that the country has been paying growing attention to this field in recent years. Similarly, with the rapid Chinese economic development and increasing public demand for medical and health care, the state’s investment and funding for medicine and health are gradually increasing. This indicates that while China has been paying more attention to this field recently, with a rapidly developing economy and increasing demand for medical care and health care, the national investment and funding in medicine and health have been increasing (27, 28). However, we also observe that the number of citations and H-index in China was low compared with other countries. This result may be due to the fact that despite China’s rapid economic development, its development in the biopharmaceutical field is relatively recent, and its foundation is not so strong yet. For example, the annual treatment cost of eculizumab is as high as US$500,000. However, China has a large population base, and the per capita medical expenses covered by medical insurance are low, leading to the low clinical utilization rate of many targeted biologics. To solve this dilemma, China should strengthen cooperation and exchange with other countries to achieve high-quality research in MG.

Regarding scholars/authors: the author with the highest number of citations and H-index was Vincent Angela. This result indicates that Vincent Angela and her research team have the highest research strength and influence in this field. They are more likely than others to publish important findings that are beneficial to MG research, and depending on the different research areas of different authors, researchers can find collaborative teams more quickly and produce high-quality articles quickly (**Figure 5**). In terms of journals/periodicals, researchers can pay more attention to journals with more publications and citations and get more timely information about the frontier of MG-targeted immunotherapy, which is more conducive to the development of research and can find the most suitable journals more quickly when submitting manuscripts to avoid delays in the timeliness of research (**Figure 6**).

Reference co-citation analysis can reflect the knowledge base of this field because the co-citation relationship of references changes over time, and these changes can represent the development and evolution of a specific field to a certain extent. Therefore, based on the reference co-citation network, we can predict the historical process and future hot research content in a specific field. Among the reference analysis results (**Figure 7A**, references of MG-related studies were classified into 15 clusters), two OMG, five muscle-specific kinases (MusK) MG (MusK-MG), 11 refractory MG (RMG), and 14 Coronavirus Disease 2019 (COVID-19) vaccine-related MG were shown. COVID-19 vaccines-related MG had relevant cited references on the timeline after 2021 (**Figure 7B**).

The clinical manifestation of MG are extremely heterogeneous, and subgroup classification based on serological profile and clinical manifestation are more meaningful for individualized treatment and prognostic assessment of MG, such as the clustering labels involving “OMG,” “RMG, “ and “ MusK-MG.” These three subtypes can be divided into different subgroups by different classification methods: (1) OMG is of interest to researchers because of the risk of converting OMG to GMG. Therefore, the need to minimize its conversion to GMG with minimal drug side effects is urgently needed because once the conversion to GMG occurs, there is a risk of critical illness, which can be life-threatening and increase the medical cost and burden on patients and the economy. Therefore, researchers have done much research in this area, such as some of our team’s studies on OMG (29–32). (2) First, understand the definition of RMG: according to the 2016 international guidelines, after treatment with corticosteroids and at least two other immunosuppressive agents, The Myasthenia gravis foundation of America Post-Intervention Status (MGFA) of patients remains unchanged or worse with persistent disabling symptoms or side effects at full dose and duration of therapy (33). Based on the definition of RMG, there is no doubt that it has been a hot topic of research, as this group of patients has failed to improve despite a full course of immunosuppressive therapy and has even experienced serious side effects. Moreover, MG is a very heterogeneous disease, with different subgroups of patients having different treatments and clinical manifestation. New treatments need to be developed for different subgroups of patients to achieve the goal of precise treatment. The urgent need to develop new therapeutic approaches to solve the problems faced by patients with RMG underscores the development of new targeted biologics for different targets, with some at phases II and III clinical trials in recent years; some have even been approved for marketing (34–36). Some targeted biologics (e.g., RTX) are also recommended in the guidelines for RMG, so there is a need for further work in this area in the future (3). MusK antibodies, the most common for MG in addition to acetylcholine receptor (AChR) antibodies, have a different pathogenesis than IgG1 and IgG3-based AChR antibodies because they are IgG4 antibodies. IgG1 and IgG3-based AChR antibodies are different (37). IgG4 is likely produced by CD20-positive short-lived plasma cells, responding better to RTX treatment (38, 39).

Due to the specificity of the clinical manifestation of MusK-MG, it is mainly characterized by the involvement of muscle groups innervated by the medulla oblongata. Therefore, attention needs to be paid to this subgroup of patients: (1) More advanced antibody detection technologies need to be developed to avoid misdiagnosis and omission, delaying treatment, and causing life-threatening conditions in this patient subgroup. (2) Since its pathogenic mechanism is different from that of traditional AChR antibodies, which cannot activate the complement system to produce membrane attack complexes, novel targeted biologics need to be developed for this subgroup of patients to enable them to reach their therapeutic goals rapidly and reduce the occurrence of critical illness.

Hot research topics related to MG and COVID-19 studies include (1) The effect of various interventions during COVID-19 pneumonia infection in patients with MG on the overall impact of MG outcome status and survival; (2) Assessment of the safety and efficacy of the COVID-19 vaccine in patients with MG; (3) Series of studies on the occurrence of MG after COVID-19 vaccination; and (4) Prognosis of MG and co-infection with COVID-19. Nevertheless, the global impact of COVID-19 is well documented. When focusing on references from the last 3 years, literature related to OMG, RMG, MusK-MG, and the relationship between the COVID-19 vaccine and MG stands out (40–43). However, compared to the overall MG research history (as shown in **Figure 8** for keyword analysis), after removing the impact of COVID-19, the research on MG itself is still biased toward clinical trials of targeted drugs for GMG and individualized treatment protocols for GMG. This suggests that for MG, a rare disease, the research is easily disturbed by many external factors, and we can choose to rub the heat of COVID-19. However, we should simultaneously assess the research closely related to MG, such as new and readily available genetic biomarkers, to select the most effective therapy for a patient or a new therapeutic target for RMG.

The results of the keyword co-occurrence analysis (**Figure 8A**) combined with the keyword burst analysis (**Figure 8B**) ultimately targeted three possible research hotspots: (1) Individualized management; (2) Randomized Controlled Trials (RCT); and (3) Targeted immunotherapy.

MG is a heterogeneous disease with differences in onset age, serological profiles, clinical manifestation, and thymoma comorbidities. The significant heterogeneity of MG leads to different clinical manifestations and treatment ideas and tools for different subgroups of patients. Therefore, precise staging of patients is needed to achieve precise treatment so patients can reach their treatment goals early and quickly with reduced disease burden. MG guidelines and consensus reports recommended that patients with MG be stratified into distinct subgroups, including early-onset, late-onset, thymoma, MusK low-density lipoprotein receptor-related protein 4, antibody-negative, and ocular forms of MG, helping with therapeutic decisions and prognosis evaluation. For instance, thymectomy is advantageous in early-onset MG but not indicated for MusK-MG. Patients with MusK-MG appear to respond better to RTX than others, whereas patients with thymoma-related MG need oncologic assessment and more prolonged and even life-long immunosuppressive therapy. Meanwhile, the efficacy of immunosuppressive treatments may vary for different gene mutation types; thus, finding new biomarkers for precise stratification at the biochemical index level is one of the hot research elements in the future. Precision therapy is currently being proposed and advocated in the field of MG and other autoimmune diseases, which are gaining widespread attention and will be the direction of future medical research.

There was few individualized well-controlled study of pharmacological and non-pharmacological interventions. Data from prospective, blinded, controlled studies were scarce, and comparisons between different treatments were lacking, thus treatment strategies for MG were primarily determined based on clinical experience. However, biologics on various targets for treating MG are in continuous development, and related basic/clinical experiments are also in progress in an orderly manner. Several biologically targeted drugs have been used in the clinical treatment of MG (e.g., RTX was the first biologically targeted drug for MG in the last 20 years, and it has been widely used in the clinic with sufficient dosing data and long-term safety data), but due to the lack of double-blind, high-quality, evidence-based medical evidence, none has been approved for use in MG. So the safety and efficacy of RTX has been a hot topic of concern, and conducting RCTs became an area of primary concern for researchers until the publication of an RCT of RTX (44, 45), which provided high-grade evidence-based medical evidence for clinical application. Furthermore, the successful publication of the REGAIN study in 2017 (34) laid the foundation for the first time for the safety and efficacy of targeted biologics in MG, thus igniting the enthusiasm for research. Second, more clinical studies with high-quality, evidence-based medicine are underway. Over time, the safety and efficacy of more targeted biologics will be established, thus encouraging researchers to turn their attention to the study of more biologics with new targets in the future, which will lay a solid foundation for the precise treatment of MG.

The disease is well-controlled in most patients, but approximately 10% fail to respond adequately to the current therapies and remain with the treatment-refractory disease. In addition, the incidence and prevalence of MG are increasing globally, particularly in older individuals, and the need to avoid the use of corticosteroids, or at least significantly reduce their use, is still unmet. Such a need is not limited to refractory patients but should concern all patients. Most recently, biologics targeting compounds of the immunological system, such as B cells (46–49), pro-inflammatory cytokines (50, 51), and their receptors (52, 53), complement system (54), and Fc neonatal receptor (55–57), are emerging as promising critical therapeutic tools to provide faster symptoms remission and better corticosteroid-sparing effects than conventional treatments. It is noteworthy that RTX can be tried in patients with MG who have failed to respond to glucocorticoid and conventional immunosuppressive drug therapy (Class IV evidence), especially in patients with MusK-MG (58). New classes of drugs have entered clinical trials and reached Drug Agencies’ authorization. These new biologics will open a new era in the field of MG treatment.

Cancer immunotherapy has been a substantial breakthrough for treating patients with a variety of malignancies second to surgery, radiotherapy and chemotherapy. The most commonly used class of cancer immunotherapy is immune checkpoint inhibitors (ICI) including block cytotoxic T-lymphocyte-associated protein 4 (CTLA-4), programmed cell death protein 1 (PD-1), or programmed death-ligand 1 (PD-L1). However, ICIs can cause severe neurological complications including encephalitis, seizure, leukoencephalopathy, myelopathy, polyneuropathy, MG and myositis (59). Previous reports described clinical characteristics (59) of ICIs- MG. Unlike classical MG, ICIs-MG is a life-threatening complication and associated with high mortality. The description of the characteristics and treatment of ICIs-MG can increase the vigilance of the clinicians and ensure the timely identification and treatment of this condition. In addition, a study (59) reported the development of MG in 12 of 9,869 cancer patients on nivolumab (PD-1 inhibitors) and none of 408 patients on ipilimumab (CTLA4 inhibitor) during the same period. Study published in 2019 (60) retrospectively collected a database of patients with cancer and MG under the treatment with ICIs. Seventy-three patients were ultimately identified, 13 of whom had MG prior to ICIs. The results of this study showed that ipilimumab did not cause MG, whereas PD-1 inhibitors can lead to ICIs-MG, that is why nivolumab was included in the keyword analysis. This is one of the hot topics in the field of MG, and the pathogenesis of mechanism of ICIs-related MG needs to be further explored in the future.

### 4.1 Limitations

The limitations of this study include: (1) literature from other databases was not included (Pubmed, Embase, Scopus, etc.). (2) Recently published literature was not included, potentially causing bias. (3) Non-English literature was included. (4) VOSviewers and CiteSpace do not provide advanced statistical analysis functions, which may introduce statistical bias. Despite these limitations, this article can still effectively describe the global trend of MG.

## 5 Conclusions

We performed a bibliometric analysis of 9,970 original MG-related articles published between 1966 and 2022, including the top five countries, institutions, scholars, journals, references, and keywords of original MG-related articles, collaborative analysis of top sections, bipartite graph overlay of journals, co-citation and clustering analysis of references, and co-occurrence and burst citation analysis of keywords. Eventually, the future research hotspots of MG were found to include two major parts: (i) research directly related to MG disease: clinical trials of various targeted drugs; the relationship between biomarkers and treatment decisions, pathogenesis, and outcome events, ultimately serving to individualize management or precision therapy; and (ii) research related to MG and COVID-19: infection with different variants of COVID-19 (e.g., Omicron) on adverse outcome events in MG; assessment of the safety of different COVID-19 vaccinations for different subtypes of MG.

## Data availability statement

The original contributions presented in the study are included in the article/**Supplementary Material**. Further inquiries can be directed to the corresponding author.

## Author contributions

Conceptualization: TC, YS, and FJ; Methodology: FJ and YS; Data analysis: FJ and YS; Software: FJ; Writing original manuscript: FJ; Review and revising manuscript: TC, YS, and FJ; Funding acquisition: TC. All authors contributed to the article and approved the submitted version.

## Supplementary material

Supplementary Material 1 Detailed report of reference co-citation and clustering analysis.

Supplementary Material 2 The Compressed package of text file with the 9,970 MG-related articles included.
